# Astrocytic function is associated with both amyloid-β and tau pathology in non-demented *APOE ϵ4* carriers

**DOI:** 10.1093/braincomms/fcac135

**Published:** 2022-05-22

**Authors:** Nicola Spotorno, Chloé Najac, Erik Stomrud, Niklas Mattsson-Carlgren, Sebastian Palmqvist, Danielle van Westen, Itamar Ronen, Oskar Hansson

**Affiliations:** Clinical Memory Research Unit, Department of Clinical Sciences, Malmö, Lund University, Lund, Sweden; Department of Radiology, Leiden University Medical Center, Leiden, The Netherlands; Clinical Memory Research Unit, Department of Clinical Sciences, Malmö, Lund University, Lund, Sweden; Memory Clinic, Skåne University Hospital, Malmö, Sweden; Clinical Memory Research Unit, Department of Clinical Sciences, Malmö, Lund University, Lund, Sweden; Department of Neurology, Skåne University Hospital, Lund University, Lund, Sweden; Wallenberg Center for Molecular Medicine, Lund University, Lund, Sweden; Clinical Memory Research Unit, Department of Clinical Sciences, Malmö, Lund University, Lund, Sweden; Memory Clinic, Skåne University Hospital, Malmö, Sweden; Image and Function, Skane University Hospital, Lund, Sweden; Diagnostic Radiology, Institution for Clinical Sciences, Lund University, Lund, Sweden; Department of Radiology, Leiden University Medical Center, Leiden, The Netherlands; Clinical Memory Research Unit, Department of Clinical Sciences, Malmö, Lund University, Lund, Sweden; Memory Clinic, Skåne University Hospital, Malmö, Sweden

**Keywords:** astrocytes, amyloid-β, APOE, myo-inositol, Alzheimer’s disease

## Abstract

A growing body of evidence suggests that astrocytes play a major role in the pathophysiology of Alzheimer’s disease. Given that *APOE* is primarily expressed in astrocytes, these cells might be an important link between the *APOE* ε4 allele and the development of Alzheimer’s disease pathology. Here, we investigate this hypothesis *in vivo* by measuring myo-inositol, a metabolite involved in astrocytic functions, with magnetic resonance spectroscopy. ﻿Currently, there is conflicting evidence regarding the relationship between *APOE* ε4 and myo-inositol concentration. Furthermore, data supporting a relationship between *APOE* ε4, myo-inositol and Alzheimer’s disease pathology (amyloid-beta and tau proteins) in the preclinical stage of Alzheimer’s disease are limited. A previous study revealed differences in myo-inositol levels between *APOE* ε4 carriers and non-carriers already in preclinical Alzheimer’s disease participants. However, other reports showed no impact of *APOE* genotype on the association between myo-inositol and the rate of amyloid-beta accumulation. In the present study, we determined the effect of *APOE* genotype on the association between myo-inositol and both amyloid-β and tau deposition quantified by PET in 428 cognitively unimpaired elderly and patients with mild cognitive impairment from the Swedish BioFINDER-2 cohort. *APOE* genotype impacted the associations between myo-inositol and amyloid-β pathology as revealed by an interaction effect between *APOE* genotype and levels of myo-inositol (*P* < 0.001) such that higher myo-inositol concentration was related to more amyloid-beta pathology in *APOE* ε4 carriers only. A similar interaction effect was also found when investigating the effect of *APOE* on the association between myo-inositol and tau pathology (*P* < 0.01). Focusing on the *APOE* ε4 subsample, myo-inositol partially (17%) mediated the association between amyloid-beta and tau pathology (*P* < 0.05). Furthermore, in a subgroup of participants with available plasma levels of glial fibrillary acidic protein, a marker of astroglial activation and astrocytosis, we found that glial fibrillary acidic protein correlated with myo-inositol only in *APOE* e4 carriers (*APOE* ε4 carriers: *P* < 0.01; *APOE* ε4 non-carriers: *P* > 0.8), suggesting that myo-inositol might reflect an aspect of the astrocytic involvement in Alzheimer’s pathology which is specific to the impact of *APOE* ε4. Therefore, we suggest that myo-inositol is a candidate *in vivo* marker to study the impact of *APOE* ε4 on the interplay between astrocytes and the pathophysiology of Alzheimer’s disease.

## Teaser

Spotorno *et al*. measured myo-inositol (mIns), a metabolite involved in astrocytic functions, with magnetic imaging spectroscopy and determined the effect of APOE on the association between mIns and both Aβ- and tau-PET retention. The results showed that higher mIns concentration was related to more Aβ and tau pathology in APOE ε4 carriers only.

## Graphical Abstract

**Graphical abstract:**
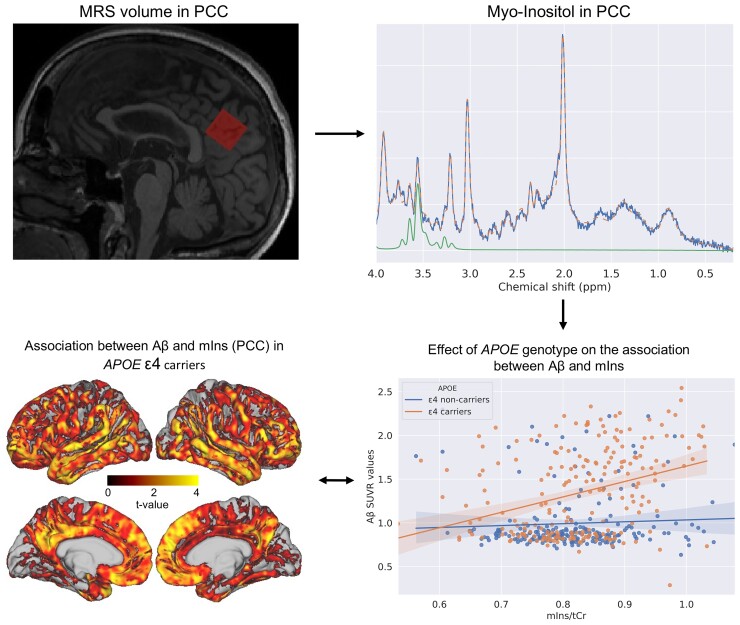

## Introduction

**﻿**Although the ε4 allele of *APOE* gene is the strongest genetic risk factor for late-onset Alzheimer’s disease, the exact mechanism mediating *APOE* ε4 pathological effect remains elusive. In the central nervous system, *APOE* is primarily expressed in astrocytes,^1^ thus the impact of *APOE* genotype on the pathophysiology of Alzheimer’s disease is likely to involve astrocytic functioning. Studies on astrocytes derived from *APOE* ε4 human-induced pluripotent stem cells have indeed shown compromised amyloid-β (Aβ) clearance^2^ as well as impairment in other basic astrocytic functions such as lipid^3^ and cholesterol^2^ metabolism, synaptic pruning^4^ and neurotrophic support^5^ when compared with *APOE* ε3 and ε2 carriers.

Mounting evidence from multiple lines of research suggests that astrocytic dysfunction has a critical impact on Alzheimer’s pathology. For example, genetic data have revealed that a significant amount of the risk for sporadic Alzheimer’s disease is associated with genes, such as Clusterin (ApoJ), Sortilin-related receptor 1, Fermitin family member 2 and *APOE*, which are primarily expressed in astrocytes.^1^ Moreover, a recent spatial transcriptomics study confirmed that genes associated with inflammatory astrocytes are among the most reactive genes in the proximity of Aβ plaques.^6^ Reactive astrocytes have also been colocalized with both Aβ plaques^7^ and neurofibrillary tangles^8^ in post-mortem studies. Aside from the neuroinflammatory cascade, it has been proposed that both the loss of the normal homeostatic function of astrocytes and the gain of toxic function could impact the clearing of Aβ from the brain via phagocytosis.^9^ Moreover, astrocytes appear to play an important role also in the tau-related pathological cascade^10,11^ with some evidence linking these phenomena to *APOE* genotype.^12^

Although multiple sources of data point to a critical role of astrocytes in the pathology of Alzheimer’s disease both independently and modulated by *APOE*, *in vivo* studies of astrocytic function in humans are still challenging. A possible approach is to use myo-inositol (mIns), a metabolite almost exclusively localized in astrocytes and measurable with magnetic imaging spectroscopy (MRS),^13,14^ as a proxy of astrocytic function. mIns is the most abundant ﻿stereoisomer of inositol^15^ and serves as a precursor molecule for inositol lipid synthesis and as a physiologically important osmolyte.^16^ Several MRS studies have previously associated mIns concentration to gliosis and astrocytic disfunctions, notably in ALS and multiple sclerosis.^17–20^ In addition, reduction in mIns levels has by associated with astrocytic damage and necrosis.^21^ In the context of the Alzheimer’s disease spectrum, multiple MRS studies have found elevated concentrations of mIns in Alzheimer’s disease patients and even in preclinical Alzheimer’s disease relative to healthy controls^22,23^ as well as an association between mIns and Aβ accumulation over time.^24,25^ However, evidence of a link between *APOE* and mIns are not conclusive, with studies reporting partially contrasting results. For example, a study from our group showed elevated levels of mIns in *APOE* ε4 carriers without evidence of Aβ accumulation when compared with non-*APOE* ε4 carriers,^22^ in-line with previous studies showing the same association in the context of healthy aging.^26,27^ However, other studies have found no effect of *APOE* genotype on the levels of mIns in healthy participants, as well as in patients from the Alzheimer’s disease spectrum.^24,28,29^

In the present study, we explored the interaction between *APOE*, astrocytes and protein aggregation focusing on non-demented individuals, including Aβ-negative cognitively unimpaired individuals, Aβ-positive cognitively unimpaired individuals (preclinical Alzheimer’s disease) and Aβ-positive patients with mild cognitive impairment (MCI) (prodromal Alzheimer’s disease). Specifically, we investigated the extent to which *APOE* modulated the association between mIns concentration and both hallmarks of Alzheimer’s disease pathology, Aβ and tau, quantified by ^18^F-flutemetamol PET and ^18^F-RO948 PET, respectively. mIns concentration was quantified in a volume located in the precuneus/posterior cingulate cortex (PCC) region, which is an early site of Aβ accumulation and has been recommended as an appropriate region for MRS in studies on Alzheimer’s disease.^30^ To support inferences about the involvement of astrocytes in the processes under examination, we also quantified plasma glial fibrillary acidic protein (GFAP), a marker of astrocytic activation, which has been shown to correlate with Aβ-PET.^31^

## Materials and methods

### Participants

Four hundred and thirty-three participants from the Swedish BioFINDER-2 study were included. Only participants with available MRS, ^18^F-flutemetamol PET and ^18^F-RO948 PET acquired within 6 months and age >50 years were included in the study cohort. To capture the entire spectrum of early Alzheimer’s disease development from subthreshold Aβ levels to abnormal Aβ levels and finally cognitive symptoms, cognitively unimpaired participants (CU–Aβ- and CU–Aβ+) and patients with MCI with evidence of Aβ pathology were included (MCI–Aβ+; see Supplementary Materials for inclusion and exclusion criteria). The data were acquired between august 2017 and October 2020 and there is no overlap with the cohort included in previous MRS studies published by our group,^22,25^ which were based on the first generation of the Swedish BioFINDER study (Swedish BioFINDER-1). Demographic and clinical characteristics are summarized in Table 1. All subjects gave written informed consent according to the Declaration of Helsinki, and the study was approved by the Ethical Review Board of Lund, Sweden.

**Table 1:** Demographic summary of the study cohort

	CU—Aβ-	CU—Aβ+	MCI—Aβ+
*N* (% female)	259 (56%)	79 (58%)	90 (48%)
Age	67 (10)	73 (9)^b^	73 (7)^b^
Years of education	13 (3)	12 (4)	13 (4)
MMSE	29.0 (1.1)	28.7 (1.3)^b^	26.5 (2.3)
*APOE* ε4 (%)	87 (34%)	58 (73%)^b^	69 (77%)^b^
Tau-positive—accordingly to tau-PET (%)^a^	0	25 (32%)^b^	49 (54%)^b^
Aβ-PET retention in the MRS volume	0.86 (0.08)	1.56 (0.33)^b^	1.73 (0.39)^b^
Tau-PET retention in the MRS volume	1.06 (0.10)	1.12 (0.20)^b^	1.23 (0.43)^b^
mIns/tCr in the MRS volume	0.80 (0.08)	0.84 (0.09)^b^	0.86 (0.11)^b^

Values are given as mean (standard deviation).

CU, cognitively unimpaired, MCI, mild cognitive impairment; Aβ+/−, amyloid-β positive/negative according to a previously published cut-off of 0.53 based on Aβ-PET^32^; MMSE, Mini-Mental State Examination; mIns/tCr, myo-inositol to total creatine ratio.

a tau positivity was based on the tau-PET retention in a medial temporal meta-ROI reflecting Braak Stage I–II, using a previously published cut-off of 1.48^33^

b significantly different from the cognitively unimpaired Aβ- group (*P* < 0.05). See Supplementary Fig. 1 for more details about differences in mIns concentrations across groups. See Supplementary results and Supplementary Fig. 2 for an alternative analysis in which participants were stratified accordingly to Aβ and tau positivity/negativity.

### Imaging protocol and analysis

#### PET protocol

^﻿^Participants underwent ^18^F-flutemetamol PET and ^18^F-RO948 PET on Discovery MI scanners (GE healthcare). ^18^F-flutemetamol PET images were acquired 90 to 110 min after injection of 185 MBq ^18^F-flutemetamol, whereas ^18^F-RO948 PET images were acquired 70 to 90 min after injection of 370 MBq ^18^F-RO948. Pre-processing and generation of ﻿standardized uptake value ratio (SUVRs) maps were carried out as previously described.^32,33^^18^F-Flutemetamol scans were normalized using the Pons as reference region, whereas ^18^F-RO948 images were referenced to the inferior cerebellar grey matter. For all subjects, Aβ status was defined based on the average SUVR values from a cortical composite using a previously published cut-off of 0.53.^32^

#### Magnetic imaging spectroscopy–MRI protocol

**Figure 1:**
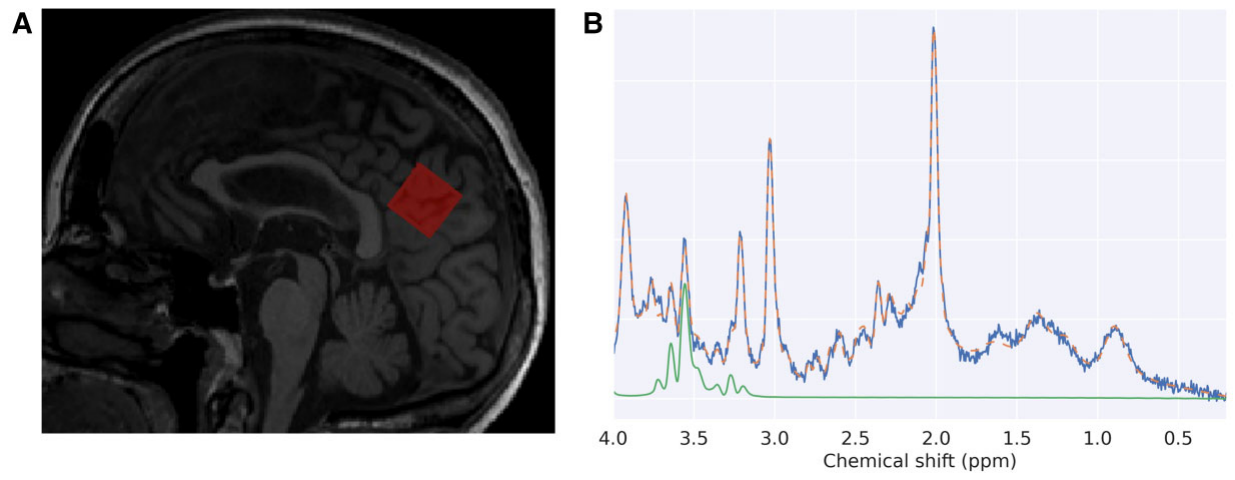
**Location of the MRS volume.** (**A**) Example of a mid-sagittal slice from one participant depicting the location of the MRS volume in red (posterior cingulate cortex/precuneus). (**B**) Example of an MRS spectra from the same participant in panel A. The blue line represents the data while the orange dashed line represents the global fit from LCmodel software. The green line depicts the specific fit of myo-inositol from LCmodel.

Both MRS and MRI data were acquired on a Siemens Prisma 3T scanner with a 64-channel receiver-coil array ﻿(Siemens Healthcare). ﻿A 2 × 2 × 2 cm^3^ MRS voxel was placed on the mid-sagittal plane in the PCC/precuneus area^30^ (Fig. 1). Single-voxel MRS data were acquired with point-resolved spectroscopy sequence with the following parameters: echo time (TE) = 30 ms; repetition time (TR) = 2000 ms; flip angle = 90 degrees; number of time domain points = 1024, spectral bandwidth = 1200 Hz. Magnetization-prepared rapid gradient-echo anatomical images were acquired with the following acquisition parameters: inversion time = 1100 ms; flip angle = 9 degrees; TE = 2.54 ms; echo spacing = 7.3 ms; TR = 1900 ms; receiver bandwidth = 220 Hz/pixel; and voxel size = 1 × 1 × 1 mm^3^. Generalized autocalibrating partially parallel acquisitions (Griswold *et al.*, 2002) was applied with acceleration factor of 2 and 24 reference lines.

#### Magnetic imaging spectroscopy analysis

Metabolite quantification was carried out using LCModel^35^ and referenced to total creatine [creatine (Cr) + Phosphocreatine (PCr) = tCr] concentration. The ratio to tCr is often used in clinical spectroscopy due to the relative stability of tCr.^36^ Five spectra were excluded from the analysis due to poor spectral quality (see Supplementary materials for the quality control procedure).

### Plasma protocol and analysis

Blood was collected in six EDTA-plasma tubes and centrifuged [2000 g, +4° (C) for 10 min]. After that, plasma was aliquoted into 1.5-ml polypropylene tubes (1 ml plasma in each tube) and stored at −80°C within 30–60 min of collection. GFAP, a marker of astroglial activation and astrocytosis, was analyzed in 288 participants using Discovery kits for HD-X (Quanterix®, Billerica, MA, USA). The analysis was performed by board-certified laboratory technicians who were blinded to clinical diagnoses.

### Statistical analysis

The relationships between demographic variables and clinical status (i.e., diagnosis) were evaluated with χ^2^ ANOVA and student *t* test (Table 1). To account for the possible confounding effect of the variable amount of grey matter present in the spectroscopic volume, mIns/tCr was modelled in a linear regression framework in the entire cohort against the fraction of grey matter in the volume (see Supplementary materials), and the residuals of this model were used for analyses. In this study, we are targeting the mIns/tCr concentration in the cortex as we measured both Aβ- and tau-PET uptake only in the cortical ribbon. Therefore, although the levels of mIns cannot be computed only from a specific tissue the proposed procedure should help in alleviating the potential confounding effect of the different amount of cortex included in the MRS volume.

The possible association between mIns/tCr ratio and PET retention were investigated using multiple regression analysis. For this purpose, the median values for Aβ-PET and tau-PET retention were derived from the same location of the MRS volume (precuneus/PCC area, see Supplementary materials for the specific procedure). Separate models were run on the full cohort including Aβ-PET or tau-PET retention as outcome (both from the location of the MRS volume), whereas mIns, *APOE* (coded as a binary value, i.e. ε4 carriers = 1, non-ε4 carriers = 0) and the interaction between the two were modelled as independent variables. The same analyses were repeated using a ﻿neocortical meta-ROI for Aβ-PET (prefrontal, lateral temporal, parietal, anterior cingulate, and posterior cingulate/precuneus) and a neocortical meta-ROI for tau-PET ﻿encompassing all stage of tau pathology (Braak I-VI).^37^ Based on previous results showing an association between plasma GFAP and Aβ accumulation,^31^ we also investigated the association between Aβ-PET retention and plasma GFAP and the potential interaction with *APOE* genotype running a similar multiple regression model using GFAP instead of mIns/tCr. We also investigated the possible association between GFAP and mIns/tCr in a linear regression framework. The interplay between Aβ, tau, astrocytic disfunction and *APOE* was further investigated by testing the potential mediation effect of mIns/tCr on the relationship between *APOE* genotype and Aβ-PET or tau-PET retention. The statistical significance of the mediation effect was estimated with bootstrapping (10 000 samples).^38^ Supplementary analyses using other commonly assessed metabolites (e.g. total N-acetylaspartate and total choline) were also performed although they were not the focus of the present report.

Sensitivity analyses in APOE ε4 carriers were also performed. First, voxel-wise maps of Aβ-PET and tau-PET retention were regressed against mIns/tCr in the PCC/precuneus. To this aim, the individual PET maps were registered to the MNI space using ANTs routines and restricted to grey matter using the previously generated mask. The voxel-wise analysis was performed with threshold-free, cluster-enhanced permutation statistics using FSL randomize (http://fsl.fmrib.ox.ac.uk/fsl/fslwiki/Randomise) with 10 000 permutations. Whole-brain statistical significance was set at the stringent family-wise error corrected threshold of *P* < 0.05. Second, considering that astrocytic dysfunction could have a significant impact on both Aβ and tau pathology, we investigated the potential mediation effect of mIns on the association between Aβ-PET and tau-PET to test whether astrocytic disfunctions contribute to the well-known relation between Aβ and tau accumulation. A further sensitivity analysis including only Aβ positive participants is reported in the Supplementary materials.

All models included age, sex and cognitive status (i.e. cognitive impaired or cognitive un-impaired) as covariates, as well as the tau-PET uptake when investigating Aβ and vice versa. A logarithmic transformation of the age variable was applied to better fit the normal distribution. Analyses were performed in Python 3.7.6 and R v3.6.1.

### Data availability

Anonymized data will be shared by request from a qualified academic investigator for the sole purpose of replicating procedures and results presented in the article and as long as data transfer is in agreement with EU legislation on the general data protection regulation and decisions by the Swedish Ethical Review Authority and Region Skåne.

## Results

### Myo-inositol concentration is associated with Aβ-PET retention in *APOE* ε4 carriers

**Figure 2:**
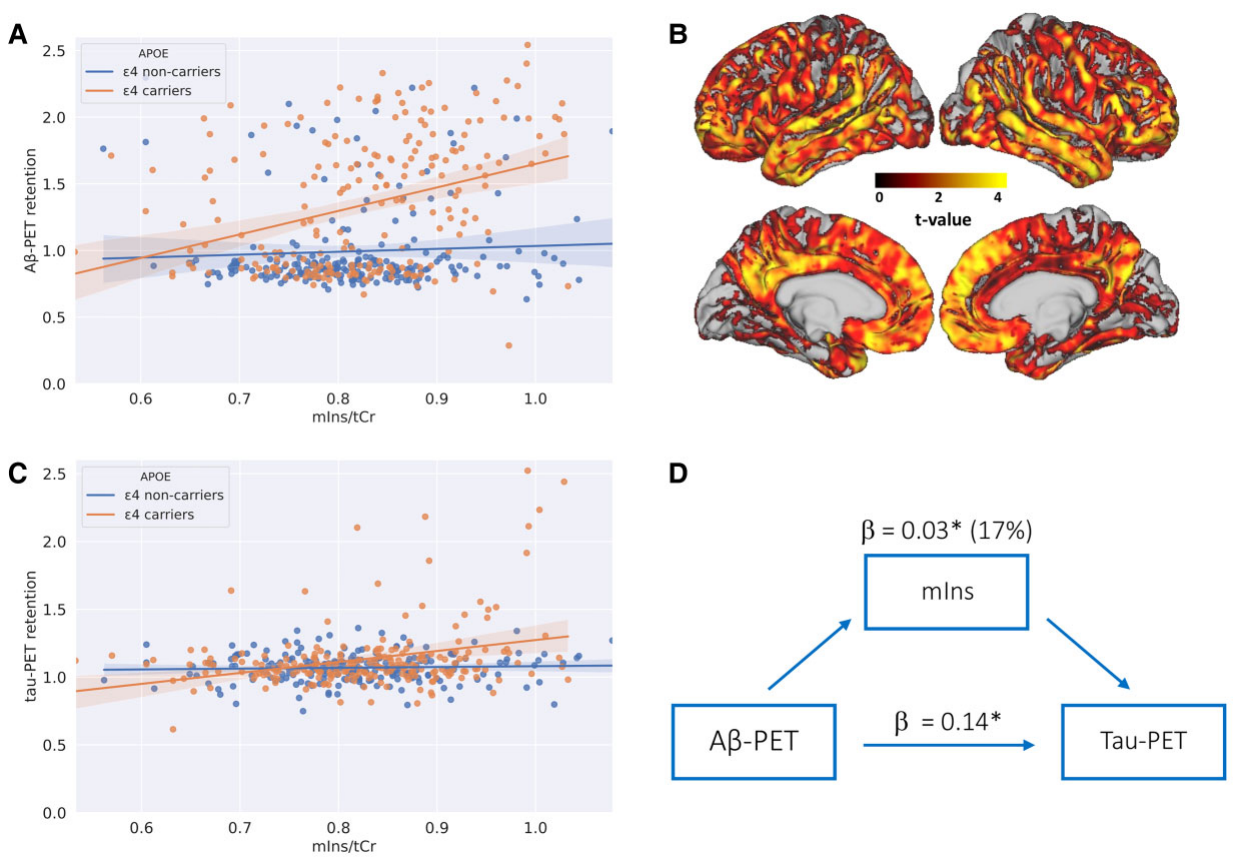
**Associations between myo-inositol and both Aβ-PET and tau-PET retention.** SUVR, standardized uptake value ratio; mIns/tCr, myo-inositol to total creatine ratio. (**A)** Co-variation of mIns/tCr and Aβ-PET retention extracted from the same location of the MRS volume. The participants were stratified in APOE ε4 carriers and non-carriers. The translucent area around the regression line represents the 95% confidential interval for the regression estimate. For visualization purposes, the ratio between mIns and tCr was depicted but the statistical analysis was performed on the residualized ratio (see the methods section; Aβ-PET ∼ mIns/tCr: *P* < 0.001 in *APOE* ε4 carriers, *P* > 0.2 in *APOE* ε4 non-carriers). (**B)** Results of the voxel-wise analysis in the *APOE* ε4 carriers: highlighted clusters represent significant (*P* < 0.05 FWE) positive correlations between Aβ-PET retention and age corrected mIns/tCr from the precuneus region. The colour scale reflects the voxel-wise *t*-values. Results were projected to surface and overlaid onto MNI (Montreal Neurological Institute) 152 template space using the connectome Workbench (v1.2 https://www.humanconnectome.org/software/connectome-workbench). (**C)** Co-variation of mIns/tCr and tau-PET retention extracted from the same location of the MRS volume (Fig. 1A). The participants were stratified in *APOE* ε4 carriers and non-carriers. For visualization purposes, the ratio between mIns and tCr was depicted but the statistical analysis was performed on the residualized ratio (see the methods section; tau-PET ∼ mIns/tCr: *P* < 0.001 in *APOE* ε4 carriers, *P* > 0.3 in *APOE* ε4 non-carriers). (**D)** Flow chart representing the mediation analysis in the *APOE* ε4 carriers. Direct effect = association between Aβ-PET and tau-PET retention extracted from the same region (precuneus/PCC): β = 0.14, *P* < 0.05; mediation effect of mIns/tCr: β = 0.03, *P* < 0.05, mIns/tCr explained 17% (β_ratio_) of the effect of Aβ-PET on tau-PET retention.

We first focused on the local association between Aβ-PET retention and mIns concentration measured in the same precuneus/PCC region. The results revealed a significant association between these two variables (model R^2^ = 0.48; mIns/tCr: β=0.36, *P* < 0.001). When introducing *APOE* genotype in the model, we found a significant interaction between *APOE* and mIns/tCr and no main effect of mIns/tCr, showing that the association between mIns and Aβ was present only in *APOE* ε4 carriers (model R^2^ = 0.54; mIns/tCr: β = −0.15, *P* > 0.5; mIns/tCr*×APOE*: β = 1.21, *P* < 0.001) (see Fig. 2A and Supplementary Table 1 for the complete statistical results). The analysis using Aβ-PET retention in a neocortical meta-ROI revealed the same results, suggesting that the association between Aβ and mIns/tCr and, most importantly, the effect of interaction with *APOE*, was not only a local phenomenon (model R^2^ = 0.54; mIns/tCr: β = −0.06, *P* > 0.4; mIns/tCr*×APOE*: β = 0.043, *P* < 0.001).

The voxel-wise analysis of the Aβ-PET data in the *APOE* ε4 carriers group provided converging evidence showing a positive association between mIns/tCr in the precuneus/PCC and widespread neocortical Aβ-PET retention, which encompassed temporal, parietal and frontal regions (see Fig. 2B). A sensitivity analysis performed in Aβ+ only participants also showed very consistent results (see Supplementary results).

Supplementary analyses of other commonly assessed metabolites (e.g., total N-acetylaspartate, and total choline) revealed no main association with Aβ and no interaction between metabolites levels and *APOE* (see Supplementary results).

### Myo-inositol concentration is associated with tau-PET retention only in *APOE* ε4 carriers

In the full cohort, we found a significant association between tau-PET retention and mIns/tCr (model R^2^ = 0.17; mIns/tCr: β = 0.41, *P* < 0.01). When including *APOE* genotype, the analysis revealed a significant interaction between mIns/tCr and *APOE* and no main effect of mIns showing that also the association between tau-PET retention and mIns/tCr was present only in *APOE* ε4 carriers (model R^2^ = 0.19; mIns/tCr: β = 0.03, *P* > 0.8; mIns/tCr*×APOE*: β = 0.79, *P* < 0.01) (Fig. 2C). When we replaced tau-PET retention from the MRS volume with tau-PET retention from the neocortical meta-ROI, the interaction between mIns/tCr and *APOE* was still significant (mIns/tCr*×APOE*: β = 0.34, *P* < 0.05). However, when we included Aβ-PET retention in a neocortical meta-ROI in the model, no significant association between tau and mIns/tCr nor an effect of interaction between mIns/tCr and *APOE* was found (model R^2^ = 0.20; mIns/tCr: β = 0.09, *P* > 0.3; mIns/tCr*×APOE*: β = 0.22, *P* > 0.1) suggesting that the association of mIns/tCr with tau, was not independent of Aβ. The voxel-wise analyses of the tau-PET maps in the *APOE* ε4 carriers group revealed no significant associations between tau-PET retention and mIns/tCr.

### Myo-inositol concentration mediate the association between Aβ-PET and tau-PET retention in *APOE* ε4 carriers

Considering the interaction effect of *APOE* on the association between mIns/tCr and both Aβ-PET and tau-PET retention, and the well-known association between Aβ-PET and tau-PET, we conducted a mediation analysis between these variables in the *APOE* ε4 carriers. The results revealed that the association between Aβ-PET and tau-PET retention, measured in the same precuneus/PCC region, was partially mediated by mIns concentration (β = 0.029, 95% CI = 0.002–0.07, *P* < 0.05, mediated effect = 17%) (Fig. 2D and the Supplementary results). In contrast, mIns/tCr does not appear to mediate the relationship between *APOE* genotype and Aβ-PET or tau-PET retention (both *P* > 0.9).

### The association between plasma glial fibrillary acidic protein levels and Aβ-PET retention is independent of APOE

**Figure 3:**
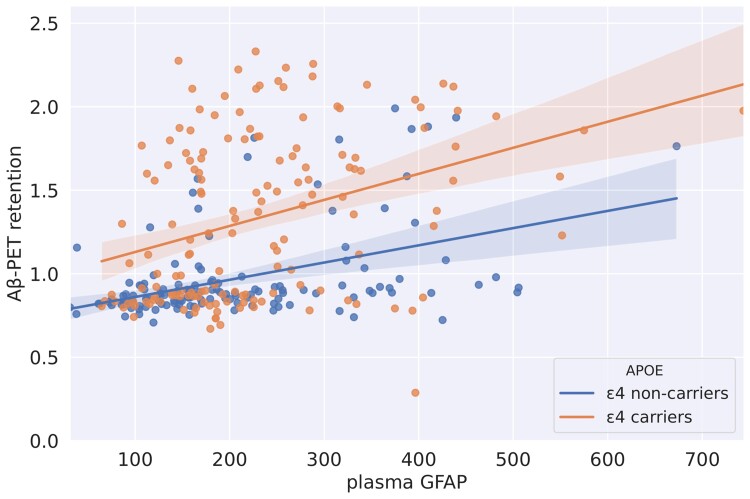
**Association between plasma GFAP levels and Aβ-SUVR values stratified by *APOE* genotype.** SUVR, standardized uptake value ratio; GFAP, glial fibrillary acidic protein. Co-variation of plasma GFAP and Aβ-PET retention extracted from the same location of the MRS volume. The participants were stratified in *APOE* ε4 carriers and non-carriers (Aβ-PET∼GFAP: *P* < 0.001 in *APOE* ε4 carriers, *P* < 0.001 in *APOE* ε4 non-carriers). The analysis was run in a subset of 288 participant with available plasma GFAP. The translucent area around the regression line represents the 95% confidential interval for the regression estimate.

In the subgroup of participants (*N* = 288: 172 CU–Aβ−; 60 CU–Aβ+; 56 MCI–Aβ+) with available GFAP plasma data a regression model showed an association of Aβ-PET retention with GFAP levels (plasma–GFAP: β = 0.0006, *P* < 0.05) but no interaction between *APOE* and GFAP level (Plasma-GFAP*×APOE*: β = 0.0005, *P* > 0.1) (Fig. 3 and Supplementary Table 3). In addition, plasma GFAP levels were significantly associated with mIns/tCr but only in *APOE* ε4 carriers (*APOE* ε4 carriers: β = 293.73, *P* < 0.01; *APOE* ε4 non-carriers: β = 16.76, *P* > 0.8).

## Discussion

The study showed that astrocytic function measured using mIns concentration in the precuneus/PCC region was positively associated with Aβ-PET retention in the same region but only in *APOE* ε4 carriers. Converging evidence from the voxel-wise analysis revealed wide-spread correlations between Aβ-PET retention in neocortical regions and mIns concentrations in the precuneus/PCC suggesting that the association between mIns and Aβ-PET was not limited to the specific location but reflected a broader relationship between mIns and Aβ. Our analyses also suggested that the link between mIns and Aβ is independent from tau accumulation. mIns also appeared to correlate with tau-PET retention only in *APOE* ε4 carriers and to partially mediate the association between Aβ-PET and tau-PET retention in such group. Interestingly, we also found an association between mIns and plasma level of GFAP but only in *APOE* ε4 carriers.

In the MRS literature, mIns is widely accepted as a glial marker. mIns has been suggested to be primarily or even exclusively present in glia and, with the highest concentrations, in astrocytes.^13,14^ mIns concentration has also been previously associated with Aβ accumulation in both cross sectional and longitudinal studies.^22,24,25,39^ However, the literature on the association between mIns and *APOE* genotype is mixed^40^ and evidence in favour or against a link between mIns, Alzheimer’s disease-related protein accumulation and *APOE* are even scarce.^40^ The present study provided data supporting of a clear link between *APOE* ε4, mIns and Aβ, from a large cohort of participants including prodromal and preclinical patients.

The lack of a mediation effect of mIns on the relationship between *APOE* and Aβ, could suggest that mIns reflected astrocytic response downstream to Aβ misfolding that is exacerbated by the *APOE* ε4 genotype. The significant mediation effect of mIns on the association between Aβ and tau mIns further suggested that such astrocytic response in *APOE* ε4 carriers has a deleterious effect on tau accumulation. ﻿Several *in vitro* studies showed that exogenously applied *APOE* ε4 has more pronounced pro-inflammatory activity compared with *APOE* ε3 in astrocytes and microglial cells,^41,42^ which could significantly affect the Aβ-related inflammatory response.^43^ This specific link is only one of the possible mechanisms that could bring together *APOE*, mIns and protein accumulation. For example, mIns plays an important role in various cellular processes as the structural basis for secondary messengers, including inositol triphosphates and phosphatidylinositol phosphate lipids^44^ which are involved in phagocytosis.^45^ Therefore, different levels of mIns could reflect a deficit in the clearing system of protein aggregates.

A further interesting result of the present study is the differential effect of *APOE* genotype on mIns concentration and plasma levels of GFAP. Although both mIns and GFAP have been considered to be markers of astrocytic activation, the two markers correlated to one another only in APOE ε4 carriers. Moreover, *APOE* did not appear to have a significant impact on the association between GFAP and Aβ. This result is in line with neuropathological evidence showing no difference in the quantification of reactive (GFAP-positive) astrocytes between APOE ε4 carriers and non-carriers.^46^ This observation should be confirmed by further studies, but it is intriguing to speculate that mIns and plasma GFAP might reflect different aspects of the astrocytic involvement in Alzheimer’s pathology.

The results presented here are not completely in line with results from previous studies. For example, Nedelska and colleagues^24^ found that *APOE* genotype did not alter the association between baseline mIns and Aβ accumulation over time. Similar results were found by ﻿Voevodskaya et al.^25^ although in this study, the authors did not model an interaction between APOE and mIns. The design of the two studies significantly differed from the design of the present study that was focus on cross-sectional analysis. Moreover, we acquired data using with a different generation of MRI scanner, which might have increased the sensitivity to subtle effects. The proportion of *APOE* ε4 carriers also differed across studies ranging from the 49% of the present study to the 29% of Nedelska *et al*^24^ and 37% of Voevodskaya *et al.*^25^. Such difference could also have had an impact on the sensitivity to the effect of *APOE* on the association between mIns and Alzheimer’s disease pathology.

Some limitation should be considered when interpreting the results of the study. A longitudinal design would be needed to test the temporal dynamic of the interplay between astrocytic response and both Aβ and tau accumulation and the impact of *APOE* genotype on such dynamic. In addition, the number of *APOE* ε4 homozygotes was too small to perform a dose-dependent analysis, whereas the number of *APOE* ε4 copies could have an important impact on the relationships we tried to elucidate. One of the novelties of the study is the comparison between mIns and the plasma level of GFAP as markers of astrocytic activity, but GFAP concentrations were not available for the entire cohort. However, the subgroup analysis including GFAP was still performed in a group of 288 participants. The lack of significant results in the voxel-wise analysis of the association between mIns and tau, also warrants further investigations. This negative result suggests that the association found at the regional level reflected a local phenomenon that did not generalize to other regions. However, this interpretation should be tested by sampling mIns levels in multiple volumes. It is also important to notice that, considering we are focusing on the early stages of the disease process, the amount of tau accumulation is still limited. This intrinsic limitation of the design could have affected our ability to detect associations between tau mIns and *APOE* genotype.

MRS is a non-invasive and inexpensive technique that can be easily applied in both research and clinical setting. The current results suggest that the quantification of mIns levels could provide a preferential astrocytic marker reflecting some of the impact of *APOE* ε4 allele on the pathophysiology of Alzheimer’s disease.

## Supplementary Material

fcac135_Supplementary_Data

## Abbreviations

Aβ =: amyloid-beta
ApoE =: apolipoprotein E
*APOE* =: gene encoding the ApoE protein
GFAP =: glial fibrillary acidic protein
mIns =: myo-inositol
PCC =: posterior cingulate cortex
SUVR =: standardized uptake value ratio
tCr =: total creatine
