# Supplementary material for: Astrocytic function is associated with both amyloid-β and tau pathology in non-demented APOE ϵ4 carriers
Source: Brain Commun. 2022 May 22;4(3):fcac135. doi: 10.1093/braincomms/fcac135 (PMC9185373; doi:10.1093/braincomms/fcac135)
Supplement: fcac135_Supplementary_Data [file fcac135_supplementary_data.docx]

**Supplementary materials**

**Materials and methods**

**Inclusion and exclusion criteria for the in the participants**

The Swedish BioFINDER-2 study enrolls participants in five sub-cohorts; Cohort A and B includes neurologically and cognitively healthy controls. The inclusion criteria are: i) ages 40-65 years (cohort A) and ages 66-100 years (cohort B); ii) absence of cognitive symptoms as assessed by a physician with special interest in cognitive disorders; iii) MMSE score 27-30 (cohort A) or 26-30 (cohort B) at screening visit; iv) do not fulfill the criteria for MCI or any dementia according to DSM-5 ^1^; v) fluent in Swedish.

Cohort C comprises participants with subjective cognitive deficits (SCD), or mild cognitive impairment (MCI; defined as a performance of <-1.5 SD below reference mean in at least one cognitive domain, see ^2^ for further details). Inclusion criteria are: i) Age 40-100 years; ii) referred to the memory clinics due to cognitive symptoms; iii) MMSE score of 24 – 30 points; iv) does not fulfill the criteria for any dementia (major neurocognitive disorder) according to DSM-5, v) fluent in Swedish.

Cohort D consists of participants with dementia due to AD. Inclusion criteria are: i) Age 40-100 years; ii) referred to the memory clinics due to cognitive symptoms; iii) MMSE score of > 12 points; iv) fulfill the DSM-5 criteria for dementia (major neurocognitive disorder) due to Alzheimer’s disease^1^; v) fluent in Swedish.

Cohort E covers other non-AD dementias and neurodegenerative disorders. Inclusion criteria are: i) Age 40-100 years; ii) fulfillment of criteria for dementia (major neurocognitive disorder) due to FTD, PDD, DLB or subcortical VaD accordingly to the DSM-5 alternatively the criteria for PD^3^, PSP^4^ , MSA^5^, CBS^6^ or svPPA^7^; iii) fluent in Swedish.

Exclusion criteria for all sub-cohorts are: i) significant unstable systemic illness that makes it difficult to participate in the study; ii) current significant alcohol or substance misuse; iii) refusing lumbar puncture, MRI or PET.

The participants to the present study have been enrolled in cohort A, B and C (only people with SCD) of the BioFINDER2 study for the cognitively unimpaired (CU) group and in cohort C (only MCI) for the cognitively impaired group. All participants were assessed by physicians with expertise in dementia disorders.

**MRS analysis**

Based on visual inspection of the spectra the following thresholds were applied to filter out corrupted or unreliable data: signal to noise ratio > 15, full width at half maximum < 6 Hz. The quality control procedure leaded to the exclusion of 5 spectra.

The % of area occupied by gray matter the MRS volume was also obtained by multiplying a gray matter mask from FreeSurfer segmentation with a mask of the MRS volume which was generated with an in-house software implemented in MATLAB (v2020a, MathWorks).

**Computing Aβ-PET and tau-PET uptake from the MRS volume**

Both Aβ-PET and tau-PET individual maps were registered to the MPRAGE space with ANTs routines (v2.3.4; <http://stnava.github.io/ANTs>). The MRS volume coordinates were used to generate a binary mask, used for the extraction of Aβ-PET and tau-PET uptake within the MRS volume, from which median values for Aβ-PET and tau-PET retention were derived. Only grey matter was included by further masking the MRS volume with a grey matter mask derived from FreeSurfer tissue segmentation (v6; <http://surfer.nmr.mgh.harvard.edu/>).

**Supplementary results**

**Mediation effect of mIns/tCr on the association between Aβ-PET and tau-PET retention in the APOE ε4 carriers**

Full cohort: mediation effect: β=0.03, 95% CI=0.003-0.07, p<0.05, mediated effect=17%; direct effect (association between Aβ-PET and tau-PET when accounting for mIns): β=0.14, 95% CI=0.06-0.22, p<0.001; total effect (association between aβ-PET and tau-PET): β=0.17, 95% CI=0.08-0.26, p<0.001.

**Sensitivity analysis in Aβ+ participants**

The analysis restricted to Aβ+ participants confirmed that mIns/tCr was associated with Aβ-PET retention only in *APOE* ε4 carriers as revealed by the significant interaction between *APOE* and mIns/tCr [*APOE**mIns: β=1.42, p<0.05] and the lack of main effect of mIns/tCr [*APOE**mIns: β=-0.44, p>0.3] (see supplementary figure 3A). The voxel-wise analysis on the Aβ-PET data in the Aβ+ *APOE* ε4 carriers provided converging evidence showing a positive association between mIns/tCr in precuneus/PCC and widespread neocortical Aβ-PET retention (see supplementary figure 3B).

In contrast, no interaction between *APOE* and mIns/tCr was found when looking for an association with tau-PET retention [*APOE**mIns: β=0.55, SE=0.43, p>0.2] as well as no main effect of mIns [mIns: β=0.08, p>0.8]. However, when limiting the group to Aβ+ *APOE* ε4 carriers, the association between tau-PET retention and mIns/tCr was marginally significant [β=0.54, p=0.057].

**Analysis of N-acetylaspartate and Choline levels**

Aside from mIns, both total N-acetylaspartate (tNAA) and total Choline (tCho) are metabolites compounds that are commonly assessed in relation to neurodegenerative diseases. tNAA is a neuronal preferential compound (NAA + NAAG) and lower levels of tNAA have been associated with neurodegeneration. Levels of tCho appeared, instead, to relate to cell membrane breakdown^8,9^. In the present study the same approach employed for the analysis of mIns was applied to tNAA/tCr and tCho/tCr. The result revealed no significant correlation between age-adjusted tNAA/tCr levels and Aβ-PET [β=-0.21, SE=0.20, p>0.2] or tau-PET [β=-0.01, SE=0.11, p>0.9] and no effect of moderation of *APOE* genotype [Aβ: tNAA**APOE*-ε4, β=0.37, SE=0.28, p>0.15; tau: tNAA**APOE*-ε4, β=-0.20, SE=0.15, p>0.15]. Similar results were found for age-adjusted tCho levels [association with Aβ-PET. tCho: β=-1.48, SE=1.12, p>0.15, tCho**APOE*-ε4: β=2.03, SE=1.59, p>0.2; association with tau-PET. tCho: β=-0.17, SE=0.60, p>0.7, tCho**APOE*-ε4: β=0.90, SE=0.85, p>0.2].

**Supplementary references**

1. American Psychiatric Association. *Diagnostic and Statistical Manual of Mental Disorders*. American Psychiatric Association; 2013. doi:10.1176/appi.books.9780890425596

2. Palmqvist S, Janelidze S, Quiroz YT, et al. Discriminative Accuracy of Plasma Phospho-tau217 for Alzheimer Disease vs Other Neurodegenerative Disorders. *JAMA - J Am Med Assoc*. 2020;324(8):772-781. doi:10.1001/jama.2020.12134

3. Gelb DJ, Oliver E, Gilman S. Diagnostic Criteria for Parkinson Disease. *Arch Neurol*. 1999;56(1):33. doi:10.1001/archneur.56.1.33

4. Litvan I, Agid Y, Calne D, et al. Accuracy of clinical criteria for the diagnosis of progressive supranuclear palsy (Steele-Richardson-Olszewski syndrome): Report of the NINDS-SPSP International Workshop. *Neurology*. 1996;47:1-9.

5. Gilman S, Wenning GK, Low PA, et al. Second consensus statement on the diagnosis of multiple system atrophy. *Neurology*. 2008;71(9):670-676. doi:10.1212/01.wnl.0000324625.00404.15

6. Armstrong MJ, Litvan I, Lang AE, et al. Criteria for the diagnosis of corticobasal degeneration. *Neurology*. 2013;80(5):496-503. doi:10.1212/WNL.0b013e31827f0fd1

7. Gorno-Tempini M, Hillis A, Weintraub S, et al. Classification of primary progressive aphasia and its variants. *Neurology*. 2011;76(11):1006-1014. doi:10.1212/WNL.0b013e31821103e6

8. Tumati S, Martens S, Aleman A. Magnetic resonance spectroscopy in mild cognitive impairment: Systematic review and meta-analysis. *Neurosci Biobehav Rev*. 2013;37(10):2571-2586. doi:10.1016/j.neubiorev.2013.08.004

9. Piersson AD, Mohamad M, Rajab F, Suppiah S. Cerebrospinal Fluid Amyloid Beta, Tau Levels, Apolipoprotein, and 1H-MRS Brain Metabolites in Alzheimer’s Disease: A Systematic Review. *Acad Radiol*. 2020;(5). doi:10.1016/j.acra.2020.06.006

**Supplementary tables**

**Supplementary table 1. Association between APOE, mIns and Aβ-PET retention**

|  | β | **SE** | **t-value** | **p-value** | **CI(0.025)** | **CI(0.975)** |
| --- | --- | --- | --- | --- | --- | --- |
| **Intercept** | -1.67 | 0.47 | -3.59 | <0.001 | -2.59 | -0.76 |
| **Cognitive status** | -0.52 | 0.04 | -12.86 | <0.001 | -0.60 | -0.44 |
| **mIns/tCr** | -0.15 | 0.24 | -0.61 | >0.5 | -0.63 | 0.33 |
| ***APOE* (binary)** | 0.21 | 0.03 | 6.71 | <0.001 | 0.15 | 0.27 |
| **mIns/tCr**APOE*** | 1.21 | 0.34 | 3.54 | <0.001 | 0.54 | 1.88 |
| **Tau-PET retention** | 0.27 | 0.07 | 3.85 | <0.001 | 0.13 | 0.41 |
| **Age(ln)** | 0.67 | 0.11 | 6.17 | <0.001 | 0.46 | 0.88 |
| **Sex** | 0.04 | 0.03 | 1.28 | >0.2 | -0.02 | 0.10 |

**Supplementary table 2. Association between APOE, mIns and tau-PET retention**

|  | β | **SE** | **t-value** | **p-value** | **CI(0.025)** | **CI(0.975)** |
| --- | --- | --- | --- | --- | --- | --- |
| **Intercept** | 0.24 | 0.27 | 0.88 | >0.3 | -0.29 | 0.76 |
| **Cognitive status** | -0.09 | 0.02 | -3.70 | <0.001 | -0.13 | -0.04 |
| **mIns/tCr** | 0.01 | 0.14 | 0.07 | >0.9 | -0.27 | 0.29 |
| ***APOE* (binary)** | 0.04 | 0.02 | 2.31 | 0.02 | 0.01 | 0.08 |
| **mIns/tCr**APOE*** | 0.75 | 0.19 | 3.86 | <0.001 | 0.37 | 1.13 |
| **Aβ-PET retention** | 0.22 | 0.06 | 3.48 | <0.01 | 0.094 | 0.338 |
| **Age(ln)** | -0.004 | 0.018 | -0.225 | >0.8 | -0.039 | 0.031 |
| **Sex** | 0.24 | 0.27 | 0.88 | >0.3 | -0.29 | 0.76 |

**Supplementary table 3. Association between APOE, mIns and GFAP levels**

|  | β | **SE** | **t-value** | **p-value** | **CI(0.025)** | **CI(0.975)** |
| --- | --- | --- | --- | --- | --- | --- |
| **Intercept** | -0.49 | 0.63 | -0.78 | >0.4 | -1.73 | 0.75 |
| **Cognitive status** | -0.52 | 0.05 | -10.62 | <0.001 | -0.62 | -0.42 |
| **Plasma GFAP** | 0.0006 | 0.000 | 2.30 | <0.05 | 8.58e-05 | 0.001 |
| ***APOE* (binary)** | 0.10 | 0.08 | 1.30 | >0.15 | -0.053 | 0.258 |
| **GFAP**APOE*** | 0.001 | 0.000 | 1.44 | >0.15 | -0.000 | 0.001 |
| **Tau-PET retention** | 0.25 | 0.08 | 3.23 | <0.01 | 0.10 | 0.40 |
| **Age(ln)** | 0.37 | 0.15 | 2.47 | <0.05 | 0.08 | 0.66 |
| **Sex** | -0.01 | 0.04 | -0.35 | >0.7 | -0.09 | 0.06 |

**Supplementary figures**

**Supplementary figure 1. myo-Inositol concentrations across groups**

**
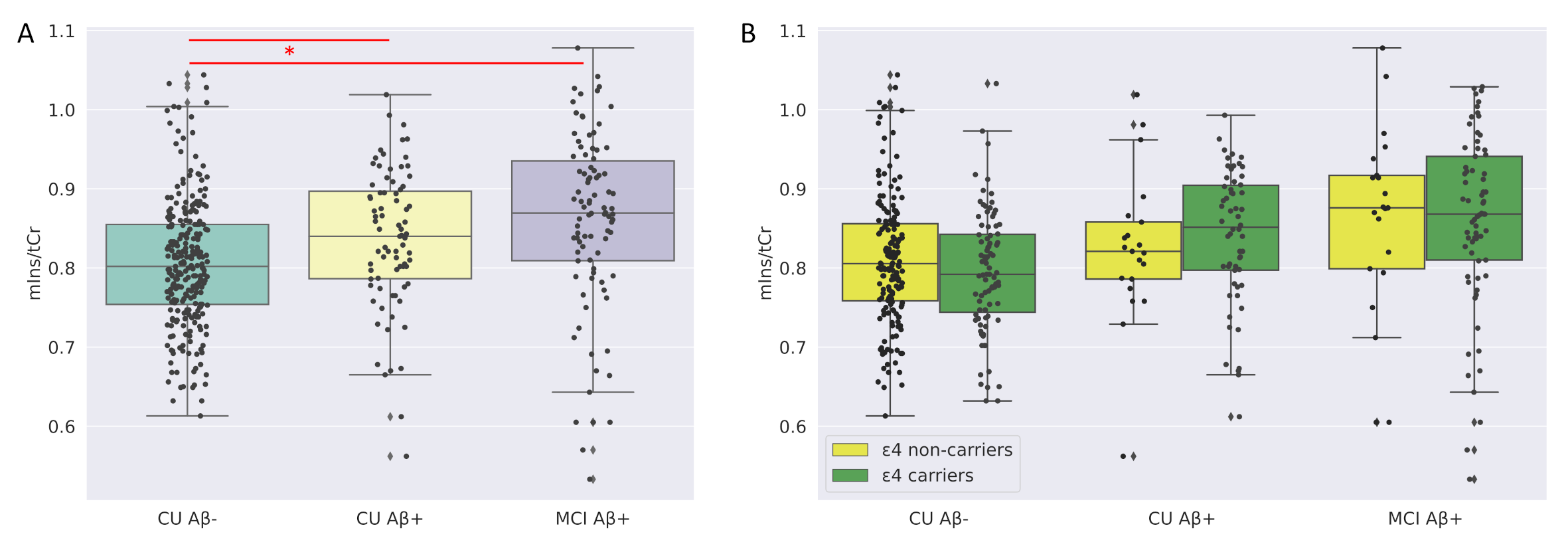
**

Abbreviations: CU: cognitively unimpaired, MCI: Mild Cognitive Impairment; Aβ+/-: amyloid-β positive/negative according to a previously published cut off of 0.53 based on Aβ-PET retention ^2^; mIns/tCr: myo-Inositol to total Creatine ratio.

**Panel A:** myo-Inositol concentration across diagnostic groups. ANOVA showed a significant difference [F=12.9, p<0.001] across groups and a Tukey post hoc test reveal that the CU–Aβ- group significantly differ from the MCI–Aβ+ group (adjusted-p < 0.01) and that the CU–Aβ- group marginally differ from the CU–Aβ+ group (adjusted-p=0.056). **Panel B:** myo-Inositol concentration across diagnostic groups stratified by *APOE* genotype. ANOVA showed no significant difference between ε4 carriers and ε4 non carriers [F=1.8, p>0.1]. the ratio between mIns and tCr was depieced for clarity but the statistical analysis was performed on the residualized mIns/tCr (see methods section).

**Supplementary figure 2. myo-Inositol concentrations across participants stratified by biomarkers**

**
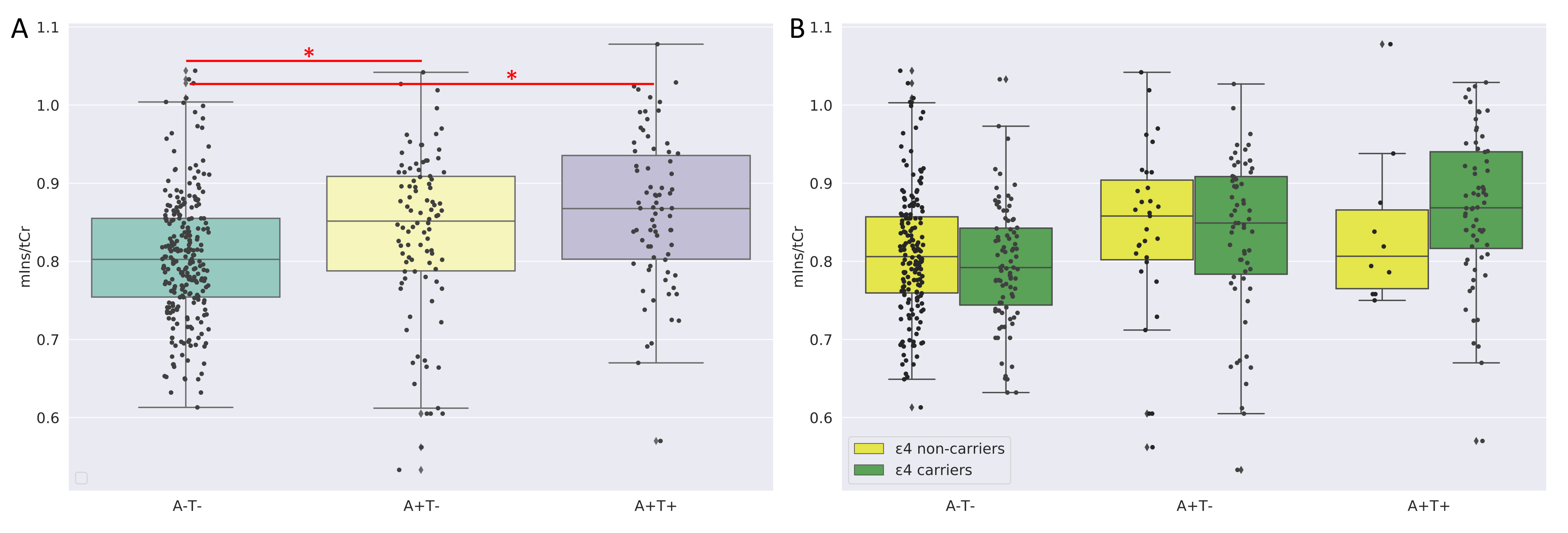
**

Abbreviations: A-T-: Aβ negative / tau negative, A+T-: Aβ positive / tau negative, A+T+: Aβ positive / tau positive; Aβ positive/negative according to a previously published cut off of 0.53 based on Aβ-PET^32^; tau positive/negative according to a previously published cut off of 1.36 based on tau-PET^33^; mIns/tCr: myo-Inositol to total Creatine ratio.

**Panel A:** myo-Inositol concentration across groups. ANOVA showed a significant difference [F=10.7, p<0.001] across groups and a Tukey post hoc test reveal that the A-T- group significantly differ from both the A+T- group (adjusted-p < 0.05) and the A+T+ group (adjusted-p < 0.01). **Panel B:** myo-Inositol concentration across diagnostic groups stratified by *APOE* genotype. No significant difference between ε4 carriers and ε4 non carriers across groups [all p>0.1]. the ratio between mIns and tCr was depieced for clarity but the statistical analysis was performed on the residualized mIns/tCr (see methods section).

**Supplementary figure 3. Associations between Aβ-PET retention and mIns/tCr in Aβ+ participants**

**
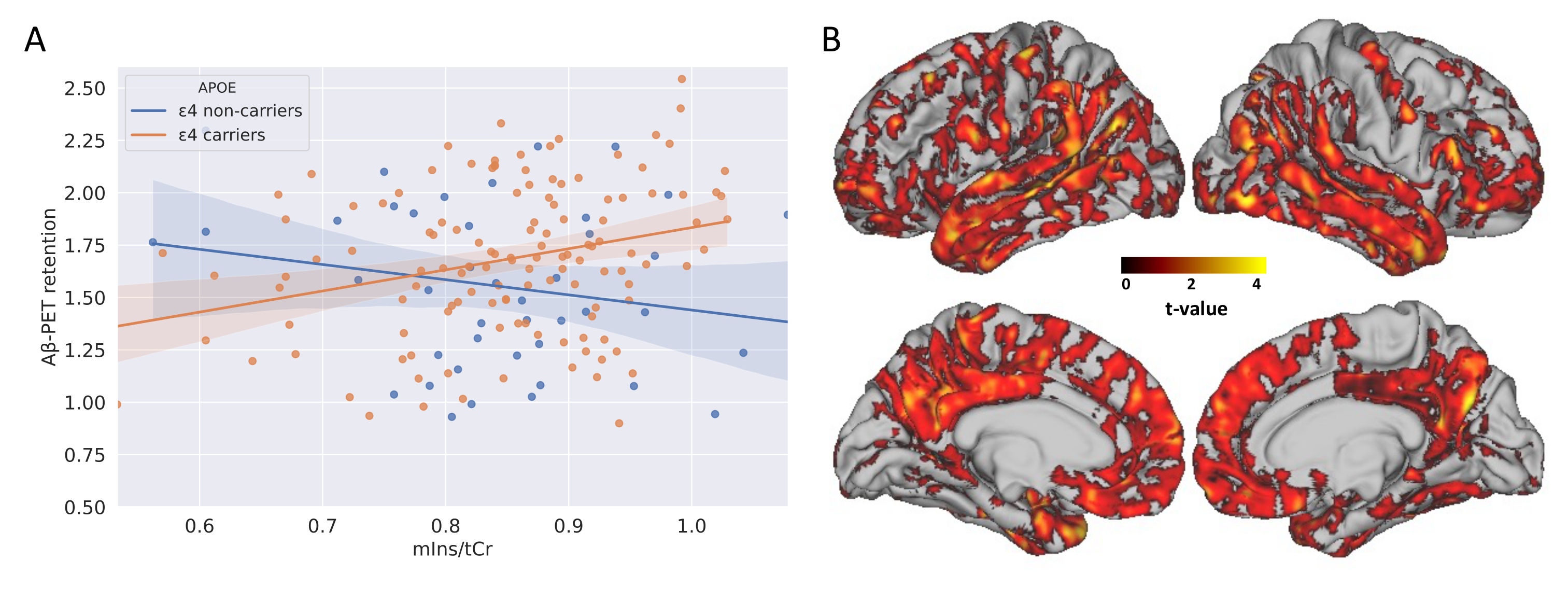
**

**Panel A:** Co-variation of mIns/tCr and Aβ-PET retention extracted from the same location of the MRS volume. The translucent area around the regression line represents the 95% confidential interval for the regression estimate. For visualization purposes the raw ratio between mIns and tCr was depicted but the statistical analysis was performed on the residualized ratio (see the methods section; Aβ-PET ~ mIns/tCr: p < 0.01 in *APOE* ε4 carriers, p > 0.4 in *APOE* ε4 non carriers). **Panel B:** Results of the voxel-wise analysis. The highlighted clusters represent significant (p<0.05 FWE) positive correlations between Aβ-PET retention and age corrected mIns/tCr from the precuneus/PCC. The color scale reflects the voxel-wise t-values. Results were projected to surface and overlaid onto MNI (Montreal Neurological Institute) 152 template space using the connectome Workbench (v1.2 https://www.humanconnectome.org/software/connectome-workbench).
